# The Effect of Telerehabilitation on Physical Fitness and Depression/Anxiety in Post-COVID-19 Patients: A Randomized Controlled Trial

**DOI:** 10.5195/ijt.2023.6560

**Published:** 2023-05-11

**Authors:** Paloma Lopes de Araújo Furtado, Maria do Socorro Brasileiro-Santos, Brenda Lopes Cavalcanti de Mello, Alex Andrade Araújo, Maria Alessandra Sipriano da Silva, Jennifer Arielly Suassuna, Gabriella Brasileiro-Santos, Renata de Lima Martins, Amilton da Cruz Santos

**Affiliations:** 1 Laboratory of Physical Training Studies Applied to Health, Federal University of Paraiba, João Pessoa, Paraiba, Brazil.; 2 Graduate Program in Physiotherapy, Federal University of Paraiba, João Pessoa, Paraiba, Brazil; 3 Associate Graduate Program in Physical Education UPE/UFPB, João Pessoa, Paraiba, Brazil; 4 Undergraduate in Kinesiology at University of Toronto, Ontario, Canada

**Keywords:** COVID-19, muscle strength, physical fitness, telerehabilitation

## Abstract

**Aim:**

The aim of this research was to evaluate the impact of a telerehabilitation program on physical fitness, muscle strength, and levels of depression and anxiety in post-COVID-19 patients.

**Methods:**

Thirty-two individuals recovered from COVID-19 (48.20±12.82 years) were allocated into either a telerehabilitation (TG n=16) or control (CG n=16) group. Physical fitness, handgrip strength, depression and anxiety levels were assessed before and after an 8-week intervention.

**Results:**

There was a significant improvement in muscle strength in both groups. Physical fitness significantly increased compared to the CG at the end of the intervention. Levels of anxiety and depression significantly decreased after the intervention when compared to the CG.

**Conclusion:**

Eight weeks of functional telerehabilitation training is a viable and efficient way to rehabilitate patients affected by COVID-19, as it improved physical conditioning and mental health.

Individuals affected by the COVID-19 disease experience cardiorespiratory, neuromuscular, and psychological dysfunction. This dysfunction persists even among those who have recovered from the disease, ultimately affecting quality of life (Li et al., 2020; Liu et al., 2020; Sheehy, 2020). A recent systematic review and meta-analysis found that persistent symptoms such as fatigue and exercise intolerance occur in approximately 80% of adult patients and that these symptoms may continue for up to 6-7 months after initial infection (Lopez-Leon et al., 2021; Wiersinga et al., 2020).

Few studies have considered the impact of physical rehabilitation on COVID-19 recovery. Liu et al. (2020) evaluated individuals with COVID-19 and was among the first studies to find an increase in the distance covered in the 6-minute walk test and improved lung function after 12 rehabilitation sessions over a six-month period. The presence of muscle pain, fatigue, and muscle weakness, as well as myopathies have been reported regardless of disease severity (Li et al., 2020; Paneroni et al., 2021).

The COVID-19 pandemic has increased measures of psychological trauma which may persist for some time, even after the spread of the virus is controlled (Xiang et al., 2020). A literature review evaluating the psychological impacts of quarantine reported that the most common negative effects of social isolation were depression, psychological distress, and increased stress levels (Brooks et al., 2020). Therefore, those individuals who have managed to overcome the effects of the virus may benefit from participation in a rehabilitation program to restore their cardiorespiratory, neuromuscular, and psychological function. Access to an in-person format is, however, necessarily limited by social distancing measures.

Telerehabilitation programs have proven to be effective and a telerehabilitation program may be a safe and viable intervention to facilitate recovery in patients after diagnosis with COVID-19. Studies that have evaluated the effects of telerehabilitation in post-COVID-19 patients have observed significant improvements in functional capacity (Amaral et al., 2022; Rodriguez-Blanco et al., 2022) and quality of life (Dalbosco-Salas et al., 2021; Li et al. al., 2022).

We decided to evaluate the effect of an eight-week telerehabilitation training program on physical fitness and on levels of depression and anxiety in individuals affected by COVID-19.

## Methods

### Study design

This is a randomized controlled clinical trial and experimental procedures adhered to guidelines published by the Consolidated Standards of Reporting Trials (CONSORT). This study was approved by the Ethics and Research Committee on Human Beings of the Federal University of Paraiba, under protocol CAEE no 44672121.8.0000.5188 and by the Brazilian registry of clinical trials (REBEC: RBR-8jgpmjf), taking on the following requirements established by Resolution 466/12 of the National Health Council and the *Declaration of Helsinki*.

### Patients and Randomization

The sample consisted of 32 individuals (males = 14; females = 18) clinically recovered from COVID-19 who met the following eligibility criteria: aged ≥ 18 years, recovery from COVID-19 for at least 20 days without the use of invasive mechanical ventilation (hospitalized patients) or any other physical and clinical restrictions, no concurrent participation in another rehabilitation program, and no cardiac, respiratory, or neurological disease, or chronic psychiatric disorders. The sample size was calculated to have 80% statistical power, with a two-tailed alpha level of 0.05, to detect a between-group difference.

The diagnosis of COVID-19 was established by clinical symptoms (including fever, fatigue, muscle pain, cough, dyspnea, among others) and a positive laboratory test (nasal swab) for the Sars-Cov-2 virus. Patients were classified as mild (clinical symptoms without dyspnea or respiratory failure) or severe (clinical symptoms with dyspnea or respiratory failure).

Individuals were recruited through digital media advertisements, folders and posters, television channels, and reports in digital newspapers. Patients underwent remote screening to assess the current conditions of their disease such as symptoms and time since infection, when the PCR test was performed, and other general health conditions. After this process, they were referred to an in-person evaluation at the Laboratory of Physical Exercise Studies Applied to Health. Then, patients were randomly allocated into either the Telerehabilitation Group (TG) or Control Group (CG) using random numbers (site: www.randomizer.org) and matched for sex and disease severity.

### Measures and Procedures

#### Physical Fitness

All patients performed several tests to evaluate physical fitness. Patients were asked to sit down and stand up in 30 seconds. This test involved the individual starting in a neutral seated position in the middle of a chair with their feet on the floor and arms crossed over their chest. After the verbal command “go,” they rose from the chair completely and returned to the starting seated position. Patients were encouraged to complete as many repetitions of this movement sequence as possible within 30 seconds. This test was used to assess lower limb strength. Patients also completed a flex-arm test. The individual started by sitting on a chair in a neutral position with their feet on the floor while holding a 2kg dumbbell with the dominant hand. After the verbal command “go,” they performed the maximum number of elbow flexion repetitions for 30 seconds. Participant maximal voluntary contraction (MVC, kg) was recorded using a handgrip dynamometer (Jamar® model PC5030J1, Medical Ibérica, Fuenlabrada, Madrid), in the most comfortable position for the hand, with the individual seated and their knee and elbow flexed at 90°. Patients were instructed to keep their forearm in half pronation, shoulder in an adducted position, and wrist in a neutral position while being able to move up to 30° of extension. The test consisted of a series of three MVCs, maintained for 5 seconds with a 1-minute interval between them. The average of the 3 repetitions of this task was obtained for each participant. Participants completed a “get up and go” task. Individuals started sitting on a chair and, when they received the verbal command “go,” patients were to walk as fast as possible to a cone (distance of 2.44m), turn around, and return to the starting position. Time (sec) was recorded and used to assess agility and balance. Patients also completed a six-minute walk test (6MWT). The individual walked as fast as possible for 6 minutes in an empty corridor of at least 30 meters. Every 60 seconds researchers verbally encouraged patients with the statements: “You are doing well” and “Keep up the good work.” The 6MWT was used to assess aerobic capacity.

#### Depression and Anxiety Levels

The Beck Depression Inventory was used to identify symptoms of depression. The Beck Depression Inventory is comprised of 21 items related to symptoms of depression. The score for each item ranges from zero to three, with zero being the absence of symptoms and three being the most intense symptoms of depression (Beck et al., 1961). A total score of 0 to 11 points means no or a minimal level of depression; 12 to 19 points is indicative of a light level of depression; 20 to 35 points indicates a moderate level of depression; and 36 to 63 points indicates a severe level of depressive symptoms.

The Beck Anxiety Inventory was used, which similarly consists of 21 items that quantify anxiety symptoms (Beck et al., 1988). The score for each item ranges from zero to three, with zero being the absence of symptoms and three being the severe level of anxiety. A total score of 0 to 10 points indicates an absence or minimum level of anxiety; 11 to 19 points a light level of anxiety; 20 to 30 points a moderate level of anxiety; and 31 to 63 points a severe level of anxiety.

### Interventions

The exercise training program was performed via telerehabilitation and involved 24 individual sessions with the patient at home while remotely connected with an exercise specialist. All sessions were conducted remotely via phone or computer video call and the exercise specialist maintained contact with the participant to ensure proper execution of the exercises.

The study followed all the characteristics of the multi-component training (single and bi-articular, multisegmental, multiplanar, dual-task, stabilization, integrated movements exercises) of neuromuscular efficiency. The intensity of exercise was monitored by a rating of Borg's perceived exertion scale, which during exercise was intended to reach a subjective rating between 6-8 on a 1-10 scale. The training program was structured according to the participant's physical capabilities in the following order. In the first month: during the 1st and 2nd-weeks, the development of the postural technique, mobility, and stability; during the 3rd and 4th-weeks; aerobic resistance and strength training. In the second month: during the 1st and 2nd weeks a focus on strength, agility, and speed, and on the 3rd and 4th-weeks a focus on agility, speed, and power. Training lasted 8 weeks, consisting of three weekly 45–60-minute sessions each divided into three parts:

- Part 1: 5-10 min warm-up (proprioception exercises) and 5-10 min strengthening (mobility exercises distributed by segments: ankle, hip, thorax, scapula and cervical, stability and neuromuscular activation)
- Part 2: 20-25 min of the main part (aerobic endurance, strength, agility, speed, and power exercises)
- Part 3: 5-10 min light saunter (static stretching exercises and/or light walking).

All COVID-19 patients participated in the remote lectures for 8 weeks (each 15 days) on health education related to chronic diseases, quality of life, and healthy lifestyle. The control group did not perform the physical training program.

### Statistical analysis

The Statistical Package for the Social Sciences (SPSS – 20.0) software was used for inferential and descriptive analyses. Data was presented as mean ± standard deviation (confidence interval) and the difference between means (response delta). Initially, a test of data normality (Shapiro-Wilk) and homogeneity of variances (Levene) was performed. The outcomes were compared by Student's t-test. In intra- and inter-group comparisons, paired and independent Student t-tests were used, respectively. The significance level adopted was p<0.05.

## Results

**Figure 1:**
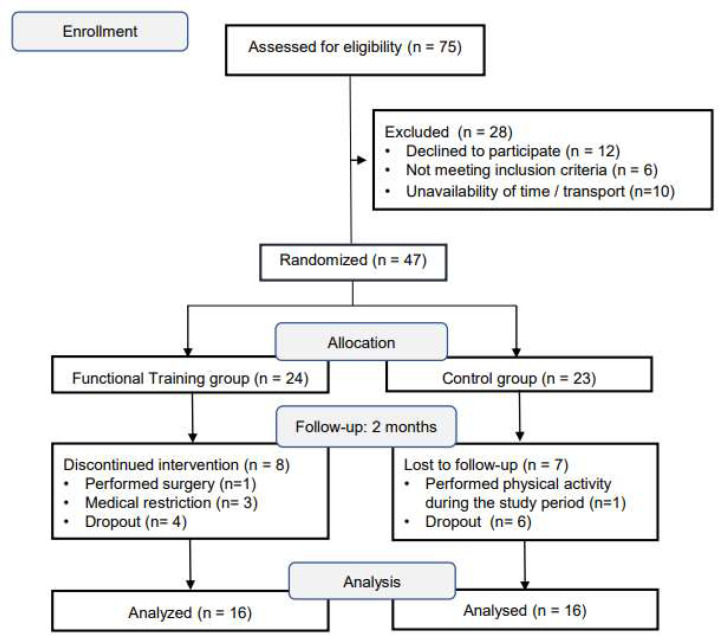
Study Flowchart CONSORT

Of 75 individuals clinically recovered from COVID-19 and initially recruited in this study, 28 were excluded because they did not meet the eligibility criteria. Forty-five individuals were evaluated and then allocated into the Telerehabilitation Training (TG n = 24) and Control (CG n = 23) groups. During the intervention, there were eight instances of attrition in the TG and seven instances of attrition in the CG, due to dropouts and medical restrictions, ending the study with 16 individuals in each group (see Figure 1).

The sample characterization data is displayed in Table 1, in which it can be observed that of the 32 individuals, 14 were male and 18 were female, with a mean age (years) of 48.35±13 years with no significant differences between the experimental and control groups. Body mass (kg) had a mean of 76.50 ± 19.32 (p=0.94), height (m) 1.63 ± 0.02 (p=0.21) and BMI (Kg/m2) 28,95 ± 6.26 (p=0.55).

**Table 1:** Characteristics of Individuals Clinically Recovered from COVID-19

Characteristics	Total	TG (n=16)	CG (n=16)
Age, years	48.35 ± 13	47.50 ± 12	49.20 ± 13
Gender (M/F), n	14M/18F	8M/8F	6M/10F
***Anthropometric***			
Weight, kg	76.50 ± 19.32	76.10 ± 20.05	76.90 ± 18.00
Height, m	1.63 ± 0.02	1.64 ± 0.01	1.61 ± 0.01
BMI, Kg/m2	28.95 ± 6.26	28.2 ± 5.90	29.70 ± 7.70
***Persistent symptoms***			
Dyspnea, (n. %)	17 (53.2)	7 (43.8)	11 (68.8)
Fatigue, (n, %)	18 (56.7)	8 (50.0)	11 (68.8)
Arthralgia, (n, %)	17 (53.2)	10 (62,5.0)	8 (50.0)
Chest pain, (n, %)	10 (31.3)	4 (25.0)	7 (43.8)
Cough, (n, %)	11 (34.3)	5 (31.2)	7 (43.8)
Anosmia, (n, %)	4 (10.0)	2 (12.5)	3 (18.8)
Rhinitis, (n, %)	3 (9.4)	2 (12.5)	0 (0.0)
Loss of Taste, (n, %)	4 (12,5)	2 (12.5)	3 (18.8)
Headache, (n, %)	16 (50.0)	8 (50.0)	8 (50.0)
Expectoration, (n, %)	3 (9.4)	2 (12.5)	2 (12.5)
Loss of appetite, (n, %)	5 (15.6)	3 (18,8)	3 (18.8)
Sore throat, (n, %)	6 (18.7)	3 (18.8)	4 (25.0)
Vertigo, (n, %)	9 (28.2)	3 (18.8)	7 (43.8)
Myalgia, (n, %)	13 (40.6)	7 (43.8)	7 (43.8)
***Clinical Classification***			
Mild, (n, %)	14 (43.7)	10 (62.5)	7 (43.8)
Severe, (n, %)	18 (56.7)	9 (56.3)	12 (75.0)
***Drug Therapy***			
Azithromycin, (n, %)	10 (31.3)	4 (25.0)	7 (43.8)
Aspirin, (n, %)	3 (9.4)	3 (18.8)	0 (0.00)
**Characteristics**	Total	TG (n=16)	CG (n=16)
Ivermectin, (n, %)	5 (15.6)	3 (18.8)	3 (18.8)
Hydroxochloroquine, (n, %)	2 (6.3)	2 (12.5)	0 (0.00)
Chloroquine, (n, %)	2 (6.3)	2 (12.5)	0 (0.00)
None, (n, %)	11 (34.3)	5 (31.2)	7 (43.8)
***Therapeutic Care***			
Hospitalization, (n, %)	15 (46.7)	7 (43.8)	9 (56.3)
Hospitalization, days	4 ± 5.3	3 ± 4.3	6 ± 5.6
O2 support, (n, %)	15 (46.7)	7 (20.0)	9 (56,3)
***Comorbidities***			
Hypertension, (n, %)	8 (25.0)	7 (43.8)	2 (12.5)
Diabetes Mellitus, (n, %)	4 (12.5)	2 (12.5)	3 (18.8)
Obesity, (n, %)	10 (31.3)	6 (37.5)	5 (31.2)

*Note*. Values are expressed in mean ± Standard Deviation; TG = Telerehabilitation Group; GC = Control Group; n = number; BMI= Body Mass Index; SAH=Systemic Arterial Hypertension. Student's t test for independent. Significance level: p<0.05.

Table 2 presents the mean values and the differences between the assessed means of the TG and CG, pre- and post-intervention for handgrip strength and physical fitness. Regarding the TG, we observed that there was a significant increase in all measures (p=0.00) when compared to pre-intervention values. When comparing the TG versus the CG, there was an improvement in fitness only in the indices of flex arm (p=0.00), sitting up (p=0.00), “get up and go” (p=0.02) and 6MWT (p=0.01). Physical fitness in the CG was not significantly different from pre-intervention (flex arm p=0.09; sitting up p=0.82; “Get Up and Go” p=0.73; 6WMT p=0.41) except for the handgrip test (p =0.00).

**Table 2:** Physical Fitness Parameters in Post-COVID-19 Patients Allocated in Telerehabilitation Group and Control Groups

Measurements	Intervention		Differences between the averages
Pre	Post
Telerehabilitation Group			
Handgrip (kg)	22.93±10 (18 – 28)	30.00±10^*^ (26 – 35)	7.16±7 (4 – 11)
Flex-arm (repetitions)	14.00±4.0 (12 – 16)	20.00±3.0^*^ (19 – 22)	6.00±4† (4 – 8)
Sitting Up – 30” (repetitions)	9.70±2.0 (9 – 11)	13.30±2.0^*^ (12 – 14)	3.60±1† (3 – 4)
“Get Up and Go” (seconds)	6.65±1.0 (6 – 7)	7.75±0.40^*^ (8 – 8)	1.00±1† (0 – 1)
6MWT (meters)	470.00±77 (432 – 508)	591.00±60^*^ (561 – 621)	122.00±64† (90 – 153)
**Control Group**			
Handgrip (kg)	22.40±8.0 (18 – 26)	28.76±11^*^ (23 – 34)	6.36±7 (3 – 10)
Flex-arm (repetitions)	15.00±5.0 (13 – 17)	17.00±5.0 (14 – 19)	2.00±3 (0 – 3)
Sitting Up – 30” (repetitions)	9.90±3.0 (9 – 11)	10.00±3.0 (8 – 12)	0.10±2 (−1 – 1)
“Get Up and Go” (seconds)	7.22±2.00 (6 – 8)	7.35±3.0 (6 – 9)	0.13±1 (−1 – 1)
6MWT (meters)	486.00±111 (432 – 541)	494.00±116 (435 – 553)	8.00±76 (−32 – 42)

*Note*. Values are expressed in mean ± Standard Deviation (Confidence Interval). 6WMT = Six-minute walk test;

* p < 0.05 = Pre vs Post, Student's t test for paired samples;

† p < 0.05 = difference between groups, Student's t test for independent samples. Significance level: p<0.05.

**Figure 2:**
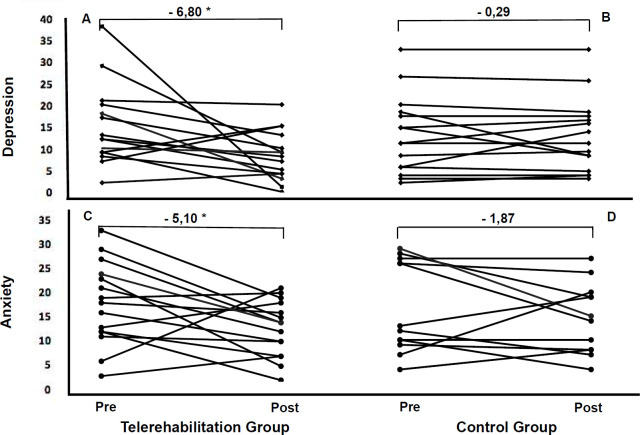
Levels of Depression and Anxiety in the Telerehabilitation Group (A and C) and Control Group (B and D), Respectively, Before and After the Intervention

*Note.* ^*^P < 0.05 = pre- vs post-intervention. Student's t-test for paired samples; Significance level: p<0.05

Regarding mental health (Figure 2), the TG showed a significant reduction in levels of depression (p=0.02) and anxiety (p=0.03) in the pre-and post-intervention comparison. However, when comparing groups, statistical differences were found for depression (p=0.00), but not for anxiety (p=0.26). In the control group, there was no improvement related to depression (p=0.67) and anxiety (p=0.10).

## Discussion

This study evaluated the effects of a telerehabilitation program on muscle strength, physical fitness, and levels of depression and anxiety among a cohort of individuals recovering from COVID-19. A significant improvement was found in all outcomes of the TG group when compared to values pre-intervention. When the TG was compared to the CG, we observed an increase in all physical fitness indices and a reduction in depression levels in the TG.

A significant increase in global muscle strength assessed by the handgrip test was found in both groups after the intervention period, even though the CG had not performed the training protocol. Interestingly, although the increase in strength was greater in the TG group when comparing the delta values of the response between the TG and the CG, they were not statistically different. These findings corroborate a study by Amaral et al. (2022), which evaluated the impact of telerehabilitation programs on cardiovascular, respiratory, and functional capabilities and concluded that there was an improvement in handgrip strength in both groups. The experimental group showed a greater increase in strength, while both groups in pre-intervention were similar (p=0.44). These findings are relevant because it is already known that handgrip strength is associated with disability, morbidity, and risk of mortality (Celis-Morales et al., 2018; Leong et al., 2015).

The flex arm and sitting-up tests have proven to be both valid and reliable instruments to assess peripheral muscle performance since they are related to physical independence. In our study, when evaluating the muscular strength of the upper and lower limbs, there was a significant improvement in muscle strength in the TG. These results corroborate those from Gonzales-Gerez et al. (2021) who evaluated patients with COVID-19 in the acute phase, with mild to moderate symptoms, undergoing a week of pulmonary rehabilitation. Compared to baseline, there was a significant increase in all outcomes in the experimental group (Gonzales-Gerez et al., 2021). In a study conducted by Cancino-López, et al. (2021), the authors observed an increase [Flex arm = 4.7 (3.7–5.9) to 8.8 (7.2–10) kilograms and sitting up = 14 (11–16) to 20 (17–21) repetitions] in muscular strength post telerehabilitation program.

The superiority of gains in physical fitness of the TG compared to the CG, more specifically in the agility observed in the stand-up and walk test, can be explained in part by the type of dynamic and challenging training that was used in the present study. Telerehabilitation training improves performance and stabilizes muscles of the spine through postural control, thereby more effectively developing the speed of performing tasks (Granacher et al., 2013).

We observed a significant increase in the distance covered by the TG compared to the CG when the aerobic capacity of the individuals was evaluated through the 6MWT. This can be explained partly by the structure format of the exercises as being of moderate-intensity and in the circuit format of the main categories of telerehabilitation training (Milanovic et al., 2015). There are few studies with physical rehabilitation after COVID-19. Amongst them, the study by Liu et al. (2020) was the first clinical trial that evaluated patients after 6 months of COVID-19 and found that 12 rehabilitation sessions increased in the distance covered and improvement in lung function. The improvement in aerobic capacity after telerehabilitation training has been recently evaluated with favorable results for the experimental group (Gonzalez-Gerez et al., 2021; Rodriguez-Blanco et., 2021; Turan et al., 2021), these authors' findings corroborate those found in our study, which showed an increase of more than 15% in the distance covered, post intervention.

Meta-analyses by Ceban et al. (2022) measured cognitive impairment from 81 studies that included COVID-19 patients; about one-third of them experienced persistent fatigue, and over one-fifth exhibited cognitive impairment after 12 or more weeks post-diagnosis. Our study showed that training was able to reduce levels of depression and anxiety when compared to pre-intervention. In addition, when comparing groups, depression levels were significantly lower in the experimental group.

These results corroborate with several studies that point out the effectiveness of rehabilitation training to improve mental health (DiLorenzo et al., 1999). Teixeira et al. (2016) found an inverse relationship between depression and physical exercise. It has been concluded that those who practiced exercise had lower levels of depression and, that as the frequency of physical activity increased, *self-esteem* levels became more accentuated while depression levels decreased.

Studies indicate that impairment of functional capacity is related to depression and is usually accompanied by stress and anxiety (Nicholson et al., 2012; Possatto & Rabelo, 2017). Because of this, it is possible to highlight the importance of physical exercise as a means to reduce the negative psychological effects after COVID-19 (lack of physical mobility, feeling of incapacity, feelings of isolation, and loneliness, amongst others).

Gordon et al. (2020) evaluated 28 healthy individuals and observed that eight weeks of resistance exercise training significantly reduced anxiety symptoms compared to the control group. Although we did not find statistically significant differences between the groups evaluated for the outcome of anxiety in our study, it is important to mention that anxiety significantly reduced in the TG and that the response in absolute values of the groups was greater for the TG, which may have clinical importance for these patients.

The main limitations of this study were the relatively small sample size, multiple tests, and the self-reported survey that can lead to type I or type II error. However, the anxiety and depression scales used in this study are validated instruments and have already been used for different populations and diseases. Another limitation could be that both groups received remote lectures on health education which may have confounded the assessment of mental health. However, this bias was minimized because both groups received the same remote lectures and content.

In conclusion, this study is the first to evaluate the association between physical performance, functional capacity, and mental health outcomes in post-COVID-19 patients. However, these results cannot be extrapolated to patients recovered from COVID-19 who were receiving invasive mechanical ventilation. Finally, eight weeks of training by telerehabilitation is an efficient way to improve the physical fitness and mental health in patients affected by COVID-19.
